# ADAM15 expression is increased in lung CD8^+^ T cells, macrophages, and bronchial epithelial cells in patients with COPD and is inversely related to airflow obstruction

**DOI:** 10.1186/s12931-020-01446-5

**Published:** 2020-07-16

**Authors:** Xiaoyun Wang, Duo Zhang, Andrew Higham, Sophie Wolosianka, Xiaoyan Gai, Lu Zhou, Hans Petersen, Victor Pinto-Plata, Miguel Divo, Edwin K. Silverman, Bartolome Celli, Dave Singh, Yongchang Sun, Caroline A. Owen

**Affiliations:** 1 Division of Pulmonary and Critical Care Medicine, Brigham and Women’s Hospital, Harvard Medical School, Boston, MA 02115 USA; 2 Program in Clinical and Experimental Therapeutics, Department of Clinical and Administrative Pharmacy, College of Pharmacy, University of Georgia, Augusta, GA 30901 USA; 3 Vascular Biology Center, Medical College of Georgia, Augusta University, Augusta, GA 30912 USA; 4 Medicines Evaluation Unit, University of Manchester, Manchester University NHS Foundation Trust, Manchester, UK; 5 Department of Pulmonary and Critical Care Medicine, Peking University Third Hospital, Beijing, China; 6 The Lovelace Respiratory Research Institute, Albuquerque, NM 87108 USA; 7 Channing Division of Network Medicine, Department of Medicine, Brigham and Women’s Hospital and Harvard Medical School, Boston, MA 02115 USA

**Keywords:** COPD, Cigarette smoke, Macrophage; CD8^+^ T cell, Epithelial cell, Forced expiratory volume in 1 s, Airflow obstruction

## Abstract

**Background:**

A disintegrin and metalloproteinase domain-15 (ADAM15) is expressed by activated leukocytes, and fibroblasts in vitro. Whether ADAM15 expression is increased in the lungs of COPD patients is not known.

**Methods:**

ADAM15 gene expression and/or protein levels were measured in whole lung and bronchoalveolar lavage (BAL) macrophage samples obtained from COPD patients, smokers, and non-smokers. Soluble ADAM15 protein levels were measured in BAL fluid (BALF) and plasma samples from COPD patients and controls. Cells expressing ADAM15 in the lungs were identified using immunostaining. Staining for ADAM15 in different cells in the lungs was related to forced expiratory volume in 1 s (FEV_1_), ratio of FEV_1_ to forced vital capacity (FEV_1_/FVC), and pack-years of smoking history.

**Results:**

ADAM15 gene expression and/or protein levels were increased in alveolar macrophages and whole lung samples from COPD patients versus smokers and non-smokers. Soluble ADAM15 protein levels were similar in BALF and plasma samples from COPD patients and controls. ADAM15 immunostaining was increased in macrophages, CD8^+^ T cells, epithelial cells, and airway α-smooth muscle (α-SMA)-positive cells in the lungs of COPD patients. ADAM15 immunostaining in macrophages, CD8^+^ T cells and bronchial (but not alveolar) epithelial cells was related inversely to FEV_1_ and FEV_1_/FVC, but not to pack-years of smoking history. ADAM15 staining levels in airway α-SMA-positive cells was directly related to FEV_1_/FVC. Over-expressing ADAM15 in THP-1 cells reduced their release of matrix metalloproteinases and CCL2.

**Conclusions:**

These results link increased ADAM15 expression especially in lung leukocytes and bronchial epithelial cells to the pathogenesis of COPD.

## Background

Chronic obstructive pulmonary disease (COPD) is currently the 3rd leading cause of death worldwide [1], and is characterized by expiratory airflow limitation that is not fully reversible and usually progressive. Airflow limitation in patients with COPD is caused both by small airway disease and pulmonary emphysema, the relative contributions of which vary between patients [2]. The most common risk factor for COPD in the developed world is smoking cigarettes [3], and this is the model used in this study.

Inhaling cigarette smoke (CS) increases oxidative stress levels in the lungs and induces alveolar septal cell apoptosis which both contribute to emphysema development [4, 5]. In addition, inhaling CS stimulates the recruitment of leukocytes into the lung, which then release proteinases that injure lung elastin and other extracellular matrix proteins and the cellular components of the alveolar walls.

Most prior studies of proteinases in the pathogenesis of COPD have focused on the contributions of serine proteinases, matrix metalloproteinases (MMPs), and cysteine proteinases [4]. However, the family of ADAM proteinases (a disintegrin and metalloproteinase domain) are emerging as key contributors to COPD as two family members (ADAM8 and ADAM9) have been recently strongly linked to COPD [6, 7]. ADAMs are multi-domain proteinases that are expressed on cell surfaces [8, 9]. ADAMs can contain a pro-domain which maintains latency of the metalloproteinase domain (MP). In addition, ADAMs can contain disintegrin and cysteine-rich domains which can regulate cell adhesion and migration, a transmembrane domain which anchors them to the cell membrane, and a short cytoplasmic domain which can regulate intracellular signaling [8, 10].

ADAM15 is expressed by monocyte/macrophage-like and lymphocyte cell lines in vitro [11], macrophages activated in vitro [12], and macrophages and fibroblasts in the joints of patients with rheumatoid arthritis [13, 14]. Macrophages, lymphocytes, and fibroblasts are thought to be involved in the pathogenesis of COPD [4]. However, with respect to other cell types linked to COPD, ADAM15 is not expressed by PMNs [15] and its expression by epithelial cells in the human lung as not been examined previously. ADAM15 has all of the domains listed above including an active MP domain which sheds growth factor receptors from cell surfaces [16]. ADAM15 also regulates cellular adhesion and migration in vitro [17, 18] and its cytoplasmic tail has been shown to bind intracellular proteins such as Src kinases [19], which are a family of non-receptor tyrosine kinases that can modulate cellular activation. However, it is not known whether the expression of ADAM15 is dys-regulated in the lungs of patients with COPD and whether this proteinase contributes to the pathogenesis of this disease. To address these gaps, we quantified ADAM15 levels in whole lung, bronchoalveolar lavage, and plasma samples from patients with COPD and matched control subjects. We also immunostained lung sections from patients with COPD and controls to localize the cells that express ADAM15. Additionally, we assessed the relationship between ADAM15 staining levels in different cell types in the lungs to severity of airflow obstruction, and pack-years of smoking history.

## Methods

### Human subjects

All studies conducted on human subjects or samples from human subjects were reviewed and approved by the institutional review boards at Brigham and Women’s Hospital and St. Elizabeth’s Hospital both in Boston, USA; Manchester University NHS Foundation Trust, Manchester, UK, and Peking University Third Hospital, Beijing, China. All subjects participating in these cohorts signed informed consent forms. Four cohorts of patients with COPD (confirmed by spirometry), smokers, and non-smokers were studied: 1) a frozen lung cohort; 2) a bronchoalveolar lavage (BAL) cohort; 3) a plasma cohort; and 4) a lung immunostaining cohort. The demographic and clinical characteristics of each cohort are shown in Tables 1, 2, 3, 4.

**Table 1:** Frozen lung Cohort: Demographic and clinical characteristics and *ADAM15* gene expression levels

Characteristics	Non-smokers^**a**^ (***N*** = 17)	Smokers^**a**^ (***N*** = 30)	COPD GOLD stages I-II (***N*** = 31)	COPD GOLD stages III-IV (***N*** = 17)	***P*** value^c^
**Number of males (%)**	12 (70)	20 (67)	22 (70)	12 (70)	***NS***
**Age (years)**	45 (20–77)	62 (25–78)	67 (50–81)	60 (49–73)	***P*** ≤ **0.02**^d^
**Pack-yrs. of smoking**	0	41 (15–80)	53 (20–100)	58 (10–114)	***P*** ≤ **0.03**^**e**^
**Number of current smokers (%)**	0 (0)	13 (43)	18 (58)	3 (18)	***P*** ≤ **0.02**^**f**^
**FEV**_**1**_**(% of predicted)**^b^	93 (79–104)	94 (65–115)	75 (51–118)	29 (10–48)	***P*** **≤ 0.008**^**g**^
**FEV**_**1**_**/FVC (% of predicted)**^b^	80 (73–84)	78 (71–89)	61 (47–68)	43 (25–60)	***P*** **< 0.001**^**h**^
***ADAM15*mRNA Levels in Lungs**	1 ± 0.6	0.84 ± 0.6	0.82 ± 0.5	2 ± 0.8	***P*** **= 0.001**^i^

The table shows the demographic and clinical characteristics of the patients with COPD, smokers without COPD, and non-smoker controls from whom lung tissue was obtained following lung volume reduction surgery, lung transplantation, a lobectomy, or a lung biopsy. Patients with COPD were sub-divided according to Global Initiative for Obstructive Lung Disease (GOLD) criteria. Total RNA was isolated from the lung samples, and *ADAM15* steady-state mRNA levels were quantified using a quantitative real-time reverse transcription PCR assay

Data are presented as median (interquartile range) for data that were not normally distributed or mean ± SD for data that were normally distributed

^a^ Non-smokers were all never-smokers. Smokers were defined as subjects who had a > 10 pack-years smoking history. Current smokers were defined as active smokers at the time the sample was obtained, or those who had stopped smoking < 1 year before the sample was obtained

^b^ All patients with COPD had forced expiratory volume in 1 s/forced vital capacity ratio (FEV_1_/FVC) < 0.7, whereas smokers without COPD and non-smoker controls had FEV_1_/FCV > 0.7

^c^ Categorical variables were analyzed with z-tests. Statistical analyses included One-Way ANOVA tests for continuous variables (age, FEV_1_% predicted, FEV_1_/FCV, and pack-years of smoking history) followed by pair-wise comparisons using 2 tailed Student’s t-tests for parametric data or Mann-Whitney U tests for non-parametric data

^d^ The non-smokers were significantly younger than the smokers, and the GOLD stage I-II and GOLD stage III-IV patients with COPD (*P* = 0.003, *P* < 0.001, and *P* = 0.02, respectively). There were no significant differences between the ages among the smoker, GOLD stage I-II patients with COPD, and GOLD stage III-IV patients with COPD groups

^e^ The pack-years of smoking histories of the GOLD stage I-II and GOLD stage III-IV COPD groups and the smoker group were significantly different from those of the non-smoker group by design (*P* < 0.001 for both comparisons). The pack-years of smoking histories of the smoker group were significantly different from those of the GOLD stage I-II and GOLD stage III-IV COPD groups (*P* = 0.005 and *P* = 0.03, respectively). The pack-years of smoking histories of GOLD stage I-II COPD group were not significantly different from those of the GOLD stage III-IV COPD group (*P* = 0.98)

^f^ The proportion of current smokers in the COPD GOLD stage III-IV group was significantly different from that of the GOLD stage I-II group (*P* = 0.02), but not the non-smoker or smoker groups (*P* = 0.2 and *P* = 0.16, respectively). The proportion of current smokers in the COPD GOLD stage I-II was significantly different from that of the non-smoker group (*P* < 0.001) but not the smoker group (*P* = 0.4). The proportion of current smokers in the smoker group was significantly different from that of the non-smoker group (*P* = 0.005)

^g^ The FEV_1_ values for the GOLD stage III-IV COPD group were significantly lower than those of the non-smoker, smoker, and GOLD stage I-II COPD groups (*P* < 0.001 for all comparisons). The FEV_1_ values for the GOLD stage I-II COPD group were significantly lower than those of the non-smoker and smoker groups (*P* = 0.008 and *P* < 0.001, respectively)

^h^ The FEV_1_/FVC ratios for the GOLD stage III-IV patients with COPD were significantly lower than those for the non-smoker, smoker, and GOLD stage I-II COPD groups (*P* < 0.001 for all comparisons). The FEV_1_/FVC ratios for the GOLD stage I-II patients with COPD were significantly lower than those for the non-smoker and smoker groups (*P* < 0.001 for both comparisons)

^i^*ADAM15* steady state mRNA levels in human lung samples expressed as fold change relative to the non-smoker control data. *P* values were adjusted to correct for differences in sex, age, pack-years of smoking history, and current smoker status between the patients with COPD and controls using an ordinal logistic regression model. After adjusting for these covariates, *ADAM15* mRNA levels in lungs samples from the patients with COPD with GOLD stage III-IV disease remained significantly higher than those in lung samples from the non-smokers, smokers, and COPD patients with GOLD stage I-II disease. The adjusted *P* value is shown in the table. *ADAM15* mRNA levels in lung samples from patients with COPD with GOLD stage I-II disease were not significantly different from those in the non-smoker and smoker samples

*NS* not significant

**Table 2:** Bronchoalveolar lavage (BAL) cohort: Demographic and clinical characteristics and ADAM15 levels

Characteristics	Non-smokers^**a**^ (***N*** = 6)	Smokers^**a**^ (***N*** = 19)	COPD Patients (***N*** = 14)	***P*** value^c^
**Number of males (%)**	5 (83)	7 (37)	7 (50)	***NS***
**Age (years)**	59 (46–73)	64 (51–75)	66 (60–74)	***NS***
**Pack-yrs. of smoking**	0	29 (12–68)	43 (7–160)	***P*** **< 0.001**^**d**^
**Number of current smokers (%)**	0 (0)	7 (37)	6 (43)	***NS***
**FEV**_**1**_**(% of predicted)**^b^	94 (83–106)	92 (71–114)	59 (10–89)	***P*** **< 0.001**^**e**^
**FEV**_**1**_**/FVC (% of predicted)**^b^	78 (74–82)	78 (72–88)	55 (41–69)	***P*** **< 0.001**^**f**^
***ADAM15*mRNA Levels in AMs (Normalized to Levels in AMs From Non-smokers)**	1 ± 0.3	1.2 ± 0.5	2 ± 0.4	***P*** **< 0.001**^g^
**ADAM15 Protein Levels in AMs (% of Levels in AMs From Non-smokers)**	100 ± 26	130 ± 27	234 ± 50	***P*** **< 0.001**^**h**^
**sADAM15 Protein Levels in BALF (pg/ml)**	7.4 (0–23)	10 (0–25)	7 (0–58)	***NS***^i^

The table shows the demographic and clinical characteristics of the patients with COPD, smokers without COPD, and non-smoker controls who underwent a bronchoscopy and bronchoalveolar lavage (BAL) as part of another research study. Patients with COPD were sub-divided according to Global Initiative for Obstructive Lung Disease (GOLD) criteria (4 subjects had GOLD stage I, 7 subjects had GOLD stage II, and 3 had GOLD stage III disease). Patients with GOLD stage IV disease were not including in the research study due to the risks associated with a bronchoscopy in this population. BAL was performed as described in Methods, and the BAL cell fraction was separated from the BALF fraction using centrifugation. BALF soluble ADAM15 (sADAM15) levels were measured using an ELISA. Alveolar macrophages (AMs) were isolated from BAL samples as described in Methods. *ADAM15* steady state mRNA levels were measured in the AM samples using a quantitative real time reverse transcription polymerization chain reaction assay and ADAM15 protein levels were measured using Western blotting and densitometry. ADAM15 mRNA and protein levels in the AMs from the smokers and COPD patients were normalized to the mRNA or protein levels, respectively, in AMs from non-smokers

Data are presented as median (interquartile range) for data that were not normally distributed or mean ± SD for data that were normally distributed

^a^ Non-smokers were all never-smokers. Smokers were defined as subjects who had a > 10 pack-years of smoking history. Current smokers were defined as active smokers at the time of the bronchoscopy or had stopped smoking < 1 year before the bronchoscopy was performed

^b^ All COPD patients had forced expiratory volume in 1 s/forced vital capacity ratio (FEV_1_/FVC) < 0.7 whereas smokers without COPD and non-smoker controls had FEV_1_/FCV > 0.7

^c^ Categorical variables were analyzed with z-test. Statistical analyses included One-Way ANOVA tests for continuous variables (age, FEV_1_% predicted, FEV_1_/FCV, and pack-years of smoking history) followed by pair-wise comparisons using 2 tailed Student’s t-tests for data that were normally distributed or Mann-Whitney U tests for that were not normally distributed

^d^ The pack-years of smoking histories of the COPD patients were not significantly different from those of the smokers (*P* = 0.177). The pack-years of smoking histories of the COPD patients and the smokers were significantly different from those of the non-smoker group by design (*P* < 0.001 for both comparisons)

^e^ The FEV_1_ values for the COPD patients were significantly lower than those of the smokers and non-smokers by design (*P* < 0.001 for both comparisons). The FEV_1_ values for the smokers were not significantly different from those for the non-smoker groups (*P* = 0.7)

^f^ The FEV_1_/FVC ratios for the COPD patients were significantly lower than those of the smoker and non-smoker groups by design (*P* < 0.001 for both comparisons). The FEV_1_/FVC ratios of the smokers were not significantly different from those of the non-smokers (*P* = 0.65)

^g^*ADAM15* steady state mRNA levels in AMs normalized to the mean value in the non-smoker group. *P* values were adjusted to correct for differences in pack-years of smoking history between the COPD patients and controls using an ordinal logistic regression model. After adjusting for these covariates, *ADAM15* mRNA levels in AMs from patients with COPD remained significantly different from those in AMs from non-smoker and smoker groups. The adjusted *P* values are shown in the Table. There were no significant differences in *ADAM15* steady state mRNA levels in AMs between the non-smoker and smoker groups

^h^ ADAM15 protein levels in AMs normalized to the mean value in the non-smoker group. *P* values were adjusted to correct for differences in pack-years of smoking histories between the COPD and control groups using an ordinal logistic regression model. After adjusting for these covariates, ADAM15 protein levels in AMs from the patients with COPD remained significantly different from those in AMs from the non-smoker and smoker groups. The adjusted *P* values are shown in the Table. There were no significant differences in ADAM15 protein levels in AMs between the non-smoker and smoker groups

^i^ Soluble ADAM15 levels in the BALF samples are shown. *P* values were adjusted to correct for differences in pack-years of smoking history between the patients with COPD and controls using an ordinal logistic regression model. After adjusting for these covariates, there were no significant differences in BALF sADAM15 levels between patients with COPD and the control groups

*NS* not significant

**Table 3:** Plasma cohort: Demographic and clinical characteristics and soluble ADAM15 levels

Characteristics	Non-smokers^**a**^ (***N*** = 28)	Smokers^**a**^ (***N*** = 27)	COPD GOLD stage I-II (***N*** = 27)	COPD GOLD stage III-IV (***N*** = 33)	***P*** value^c^
**Number of males (%)**	18 (64.3)	17 (63)	23 (85)	20 (60.6)	***NS***
**Age (years)**	66 (35–77)	62 (50–74)	67 (53–83)	65 (48–82)	***NS***
**Pack-yrs. of smoking**	0	50 (15–96)	60 (20–127)	68 (12–160)	***P*** ≤ **0.049**^**d**^
**Number of current smokers (%)**	0 (0)	3 (10)	14 (52)	7 (21)	***P*** ≤ **0.03**^**e**^
**FEV**_**1**_**(% of predicted)**^b^	98 (79–147)	91 (65–119)	65 (50–105)	35 (17–49)	***P*** **< 0.001**^**f**^
**FEV**_**1**_**/FVC (% of predicted)**^b^	77 (70–86)	76 (71–84)	57 (38–68)	39 (26–59)	***P*** **< 0.001**^**g**^
**Plasma sADAM15 Levels (pg/ml)**	665 (175–2457)	642 (256–2393)	517 (64–1110)	672 (54–3533)	***NS***^h^

The table shows the demographic and clinical characteristics of the patients with COPD, smokers without COPD, and non-smoker controls included in the analysis of plasma soluble ADAM15 protein levels. Patients with COPD were sub-divided according to the Global Initiative for Obstructive Lung Disease (GOLD) criteria

Data are presented as median (interquartile range) for data that were not normally distributed or mean ± SD for data that were normally distributed

^a^ Non-smokers were all never-smokers. Smokers were defined as subjects that had > 10 pack-year smoking history. Current smokers were defined as active smokers at the time of the biopsy or surgery or had stopped smoking < 1 year before the sample was obtained

^b^ All patients with COPD had a forced expiratory volume in 1 s/forced vital capacity ratio (FEV_1_/FVC) < 0.7 whereas smokers without COPD and non-smoker controls had a FEV_1_/FCV ratio > 0.7

^c^ Categorical variables were analyzed with z-test. Statistical analyses included One-Way ANOVA tests for continuous variables (age, FEV_1_% predicted, FEV_1_/FCV, and pack/years) followed by pair-wise comparisons using 2 tailed Student’s t-tests for parametric data or Mann-Whitney U tests for non-parametric data

^d^ The pack/year smoking histories of the GOLD stage I-II and GOLD stage III-IV COPD groups and the smoker group were significantly different from those of the non-smoker group by design (*P* < 0.001 for both comparisons). The pack/year smoking histories of the GOLD stage III-IV COPD group were significantly different from those of the smoker group (*P* = 0.049), but not significantly different from those of the GOLD stage I-II COPD group (*P* = 0.48). The pack/year smoking histories of the GOLD stage I-II COPD group were not significantly different from that of the smoker group (*P* = 0.2)

^e^ The proportion of current smokers in the GOLD stage I-II COPD patients was significantly different from that of the non-smokers, smokers, and GOLD stage III-IV COPD patients (*P* < 0.001, *P* = 0.003, and *P* = 0.03, respectively). The proportion of current smokers in the GOLD stage III-IV COPD group was significantly different from that of the non-smoke group (*P* = 0.029), but not significantly different from that of the smoker group (*P* = 0.43)

^f^ The FEV_1_ values of the GOLD stage III-IV COPD patients were significantly different from those of the non-smoker, smoker, and GOLD stage I-II COPD groups (*P* < 0.001 for all comparisons). The FEV_1_ values of the GOLD stage I-II COPD group were significantly different from those of the smoker and non-smoker groups (*P* < 0.001 for both comparisons)

^g^ The FEV_1_/FVC ratios of the GOLD stage III-IV COPD group were significantly different from those of the non-smoker, smoker, and GOLD stage I-II COPD groups (*P* < 0.001 for both comparisons). The FEV_1_/FVC ratios of the GOLD stage I-II COPD patients were significantly different from those for the smoker and non-smoker groups (*P* < 0.001 for both comparisons)

^h^Soluble ADAM15 levels in plasma samples (medians and interquartile ranges) are shown in the Table. *P* values were adjusted to correct for differences in pack-years of smoking history and current smoker status between the COPD patients and controls using an ordinal logistic regression model. After adjusting for these covariates, plasma sADAM15 levels were not significantly different between the COPD patients and controls

*NS* not significant

**Table 4:** Lung immunostaining Cohort: Demographic and clinical characteristics

Characteristics	Non-smokers^**a**^ (***N*** = 10)	Smokers^**a**^ (***N*** = 10)	COPD GOLD stages I-II (***N*** = 14)	COPD GOLD stages III-IV (***N*** = 17)	***P*** value^c^
**Number of males (%)**	4 (40)	4 (40)	9 (64)	7 (41)	***NS***
**Age (years)**	63 ± 12	65 ± 9	65 ± 10	61 ± 7	***NS***
**Pack-yrs. of smoking**	0	38 (10–84)	62 (18–120)	42 (13–80)	***P*** **< 0.001**^d^
**Number of current smokers (%)**	0 (0)	2 (20)	1 (7)	0 (0)	***NS***
**FEV**_**1**_**(% of predicted)**^b^	97 (60–133)	87 (70–108)	72 (50–115)	24 (13–46)	***P*** **≤ 0.03**^**e**^
**FEV**_**1**_**/FVC (% of predicted)**^b^	82 (74–111)	81 (72–111)	61 (40–70)	39 (20–68)	***P*** **< 0.001**^**f**^

The table shows the demographic and clinical characteristics of the patients with COPD, smokers without COPD, and non-smoker controls who underwent either a lung biopsy, lung volume reduction surgery, or lung transplantation (see Online Supplement). Patients with COPD were sub-divided according to the Global Initiative for Obstructive Lung Disease (GOLD) criteria

Data are presented as median (interquartile range) for data that were not normally distributed or mean ± SD for data that were normally distributed

^a^ Non-smokers were all never-smokers. Smokers were defined as subjects that had a > 10 pack-years smoking history. Current smokers were defined as active smokers at the time of the surgery or had stopped smoking < 1 year before the biopsy or surgery

^b^ All patients with COPD had forced expiratory volume in 1 s/forced vital capacity ratio (FEV_1_/FVC) < 0.7, whereas smokers without COPD and non-smoker controls had FEV_1_/FCV > 0.7

^c^ Categorical variables were analyzed with z-test. Statistical analyses included One-Way ANOVA tests for continuous variables (age, FEV_1_% predicted, FEV_1_/FCV, and pack-years of smoking history) followed by pair-wise comparisons using 2 tailed Student’s t-tests for parametric data or Mann-Whitney U tests for non-parametric data

^d^ The pack-years of smoking histories of the GOLD stage I-II and GOLD stage III-IV COPD groups were not significantly different from those of the smoker group (*P* = 0.1 and *P* = 0.6, respectively). The pack-years of smoking histories of the GOLD stage I-II and GOLD stage III-IV COPD groups and the smoker group were significantly different from those of the non-smoker group by design (*P* < 0.001 for all comparisons)

^e^ The FEV_1_ values of the GOLD stage III-IV patients with COPD were significantly different from those of the non-smokers, smokers, and GOLD stage I-II COPD group by design (*P* < 0.001 for all comparisons). The FEV_1_ values for the GOLD stage I-II COPD group were significantly different from those of the non-smoker and smoker groups (*P* = 0.006 and *P* = 0.03, respectively)

^f^ The FEV_1_/FVC ratios of the GOLD stage III-IV patients with COPD were significantly different from those of the non-smoker, smoker, and GOLD stage I-II COPD groups (*P* < 0.001 for all comparisons). The FEV_1_/FVC ratios of the GOLD stage I-II patients with COPD were significantly different from those of the non-smoker and groups (*P* < 0.001 for both comparisons)

*NS* not significant

In all of the cohorts, current smokers were defined as active smokers at the time of the study or smokers that had stopped smoking less than 1 year prior to the study. Smokers were defined as having COPD if they had a > 10 pack-years of smoking history and a post-bronchodilator FEV_1_/FVC ratio < 0.7. Subjects with a post-bronchodilator FEV_1_/FVC ratio > 0.7 were considered to be controls.

#### Frozen lung tissue cohort

Lung tissue was obtained from patients with COPD (Global Initiative for Obstructive Lung Disease [GOLD] spirometric stages I-IV who underwent either lung volume reduction surgery, lung transplantation, a lobectomy for cancer (Table 1). Lung tissue was obtained from smokers and non-smokers (all of whom were never smokers) who underwent a lobectomy for a tumor or a lung biopsy. In all cases in which tissue was obtained from a lung with a tumor, tissue sampling corresponded to an area > 10 cm from the tumor. None of the subjects studied had evidence of respiratory tract infection at the time of sampling. Frozen lung tissues were provided by Manchester University NHS Foundation Trust, Manchester, UK (*n* = 45 subjects), and Peking University Third Hospital, Beijing, China (*n* = 50 subjects). Lung tissue was frozen at -80 °C and then used to quantify *ADAM15* steady state messenger ribonucleic acid (mRNA) levels using quantitative real-time reverse transcription polymerase chain reaction (qRT-RT-PCR) assays as outlined below.

#### BAL cohort

Bronchoscopy was performed on 14 clinically-stable patients with COPD (GOLD stage I-III disease), 19 smokers without COPD, and 6 non-smokers without COPD (all of whom were never smokers) in a cohort recruited at Brigham and Women’s Hospital, Boston, USA as part of another research study (Table 2). Patients with COPD and GOLD stage IV disease were not including in this research study due to the risks associated with a bronchoscopy in this population with very severe disease.

The bronchoscope was wedged in the bronchus and a maximum of 4 × 60 ml aliquots of pre-warmed sterile 0.9% NaCl solution were instilled into the right and/or left upper lobes. The aspirated BAL sample was stored on ice, and then filtered using a 100 μm filter (Thermo Fisher Scientific, Carlsbad, CA). The filtrate was centrifuged (at 400 g for 10 min at 4 °C) and the BALF sample was removed and stored in aliquots at -80 °C for further analysis. The BAL cell fraction was suspended in 5 ml Ammonium-Chloride-Potassium Lysis Buffer (Thermo Fisher Scientific, Carlsbad, CA) and incubated at room temperature for 5 min to lyse red blood cells. Alveolar macrophages (AMs) were isolated from the BAL samples by adhering the cells to tissue culture dishes for 2 h at 37 °C and removing the non-adherent cells by washing the plates with sterile PBS. The AMs that were adherent to tissue culture plates were either lysed in Buffer RLT (QIAGEN, Hilden, Germany) for qRT-PCR assays to measure *ADAM15* transcripts or lysed in radioimmunoprecipitation assay (RIPA) buffer to measure ADAM15 protein levels using Western blotting.

#### Plasma cohort

Heparinized blood samples were obtained from 60 clinically-stable patients with COPD (GOLD stage I-IV disease), 27 smokers without COPD, and 27 non-smokers (all of whom were never smokers) in a cohort recruited in Boston, MA, USA (at St Elizabeth’s Hospital and Brigham and Women’s Hospital) as part of another research study (Table 3). Plasma was removed and frozen in aliquots at -80 °C and then used to quantify ADAM15 levels using an enzyme-linked immunosorbent assay (ELISA; R&D system) that recognizes the ectodomain of the protein.

#### Lung immunostaining cohort

We studied current or former smokers with COPD (GOLD stages I–IV); smoker controls without COPD, and non-smokers (all of whom were never smokers). The characteristics of the subjects studied are shown in Table 4. Sections of lung were obtained from lung biopsies obtained from subjects with benign tumors or lobectomies for lung cancer. In the lung cancer cases, lung tissue for study was taken > 10 cm from the cancer margin. ADAM15 staining was similar in the cancer and non-cancer cases in each group (data not shown). Lung tissue was also obtained from lung volume reduction surgeries, or from explanted lungs from patients with COPD undergoing lung transplantation. Lung sections were provided by the National Heart, Lung, and Blood Institute-sponsored Lung Tissue Research Consortium (www.ltrcpublic.com;*n* = 20 subjects), or the Department of Pathology at Brigham and Women’s Hospital, Boston (*n* = 31 subjects). None of the subjects studied had evidence of respiratory tract infection at the time of lung tissue sampling.

#### ADAM15 steady state mRNA levels in frozen lung and AMs from the BAL cohort

*ADAM15* steady-state mRNA levels were quantified in human frozen lung samples or AMs obtained from patients with COPD, smokers, and non-smokers. Total RNA was isolated from lung samples or AMs using a SurePrep TrueTotal RNA Purification Kit (Fisher Scientific, Fair Lawn, NJ), following the manufacturer’s instructions, and 1 μg of RNA was reverse transcribed into cDNA using a High-Capacity cDNA Reverse Transcription Kit (Thermo Fisher Scientific, Carlsbad, CA). A SYBR green-based real-time RT-PCR assay was used to measure *ADAM15* gene expression using primers for *ADAM15* (forward primer 5′- CAGGACGATCTCCCAATTAGC − 3′; reverse primer 5′- GGACCAACTCCCTATTCTGTAGC-3′) from Invitrogen (Charlestown, MA) and primers for peptidylprolyl isomerase A (*PPIA*) as the housekeeping gene (forward primer 5′- CTCGAATAAGTTTGACTTGTGTTT − 3′; reverse primer 5′- CTAGGCATGGGAGGGAACA − 3′), the comparative threshold method, and an AriaMx Real-time PCR machine (Agilent technologies, Santa Clara, CA).

#### ADAM15 protein levels in AMs from the BAL cohort

AMs were lyzed in RIPA buffer containing 1 mM phenylmethylsulfonylfluoride, 1 mM 1,10 phenanthroline, 1% Sigma Mammalian Protease Inhibitor Cocktail. The total protein concentration was measured using a BCA protein assay kit (Thermo-Fisher Scientific, Rockford, IL). Proteins in the cell lysates were separated on 12% SDS-PAGE gels, transferred to PVDF membranes, and then incubated with murine anti-ADAM15 IgG (R & D Systems, Minneapolis, MN), or murine anti-HSP90 IgG (BD Biosciences, Franklin Lakes, NJ). Membranes were then incubated with goat anti-murine IgG-conjugated to horse raddish peroxidase (Bio-Rad, Hercules, CA), and signals were developed using a chemiluminescence kit. Western blot images were captured and analyzed using a Chemidoc (Bio-Rad, Hercules, CA).

#### Double immunofluorescence staining of sections of lung from the lung immunostaining cohort for ADAM15 and markers of different cell types in the lung

Immunofluorescence staining for ADAM15 was performed on formalin-fixed sections of lung. The sections were incubated with a goat anti-human ADAM15 IgG which recognizes the ectodomain of ADAM15 (R&D Systems, Minneapolis, MN) or non-immune goat IgG, followed by Alexa 488-conjugated rabbit anti-goat fragment antigen binding-2 (F (ab)_2_; Invitrogen, Charlestown, MA). Sections were double stained with either: 1) murine anti-human Cluster of Differentiation 68 (CD68) IgG (Abcam, Cambridge, MA) followed by Alexa 546-conjugated goat anti-murine IgG; or 2) rat anti-human CD8 IgG (Abcam, Cambridge, MA) followed by Alexa 546-conjugated goat anti-rat F (ab)_2_; or 3) murine anti-human pancytokeratin (PanCK) (Sigma, St. Louis, MO) followed by Alexa 546-conjugated goat anti-murine IgG; or 4) murine anti-human α-smooth muscle actin (α-SMA; Sigma, St. Louis, MO) followed by Alexa 546-conjugated goat anti-murine F (ab)_2_; or 5) murine anti-human CD4 (R&D Systems, Minneapolis, MN) followed by Alexa 546-conjugated goat anti-murine F (ab)_2_; or 6) rat anti-human CD45R (Abcam, Cambridge, MA) followed by Alexa 546-conjugated goat anti-rat F (ab)_2_. All the secondary antibodies were obtained from Invitrogen (Charlestown, MA). Lung sections were also immuno-stained with appropriate isotype-matched non-immune control antibodies. Images were captured using an epi-fluorescence microscope (Leica Biosystems, Buffalo Grove, IL). The percentage of AMs and CD8^+^ T cells in the alveolar spaces in 10 randomly acquired images per lung section that stained positively for ADAM15 was enumerated. The number of positively stained bronchial epithelial cells, alveolar epithelial cells, or airway α-SMA-positive cells was quantified and normalized to the unit of area of the bronchial epithelium, alveolar wall, or airway wall, respectively using MetaMorph software (Molecular Devices, LLC, San Jose, CA). Cells staining positively for ADAM15 were defined as cells that had greater staining intensity than the non-specific staining that detected in the same cell type in sections that were immunostained with an isotype-matched non-immune control antibody.

#### Soluble ADAM15 protein levels in plasma and BALF samples

Soluble ADAM15 (sADAM15) protein levels were measured in plasma and BALF samples from non-smokers without COPD (all of whom were never smokers), smokers without COPD, and patients with COPD using commercially-available ELISAs (R&D Systems, Minneapolis, MN). Tables 2 and 3 show the demographic and clinical data of the subjects studied.

#### Overexpression of ADAM15 in human THP-1 cells

Human *ADAM15* cDNA (isoform 6 pre-proprotein as the longest transcript variant) amplified using RT-PCR was subcloned into the BamHI and XhoI sites of the pcDNA3.1 vector to obtain pcDNA-*ADAM15* (the primer sequences used for cloning were: forward: 5′-CGCGGTACCATGCGGCTGGCGCTGCTCTGGG-3′; reverse: 5′-CCGCTCGAGTCAGAGGTAG-AGCGAGGACACT-3′). THP-1 cells (human monocyte-like cells; ATCC, Manassas, VA) were maintained in RPMI-1640 containing 10% fetal bovine serum and differentiated into macrophage-like cells by incubating them with 150 nM phorbol myristate acetate for 24 h. Cells were electroporated (Neon Transfection System; Thermo Fisher Scientific, Waltham, MA) with either the ADAM15 or the control (pcDNA3.1) vector [20]. ADAM15 expression was measured using quantitative RT RT-PCR and the THP-1 cells were treated with 5% CSE for up to 24 h. C-C motif chemokine ligand-2 (CCL-2), tumor necrosis factor-α (TNF-α), MMP-9, and MMP-12 levels were measured in the cell culture supernatants using ELISAs (R&D Systems, Minneapolis, MN).

#### Statistics

Normally-distributed data (presented as means ± SD or SEM) were analyzed with One-Way ANOVAs followed by post-hoc testing with 2-tailed Student’s t-tests. Data that were not normally distributed (presented as box-plots showing medians and 25th and 75th percentiles and whiskers showing 10th and 90th percentiles) were analyzed with Kruskal-Wallis One-Way ANOVA followed by pair-wise comparisons using Mann-Whitney U-tests. *P* values were adjusted to correct for differences in sex, age, pack-years of smoking history, and/or current smoker status between the COPD and control groups using an ordinal logistic regression model. *P* ≤ 0.05 was considered statistically significant.

## Results

### Whole lung samples

**Fig. 1:**
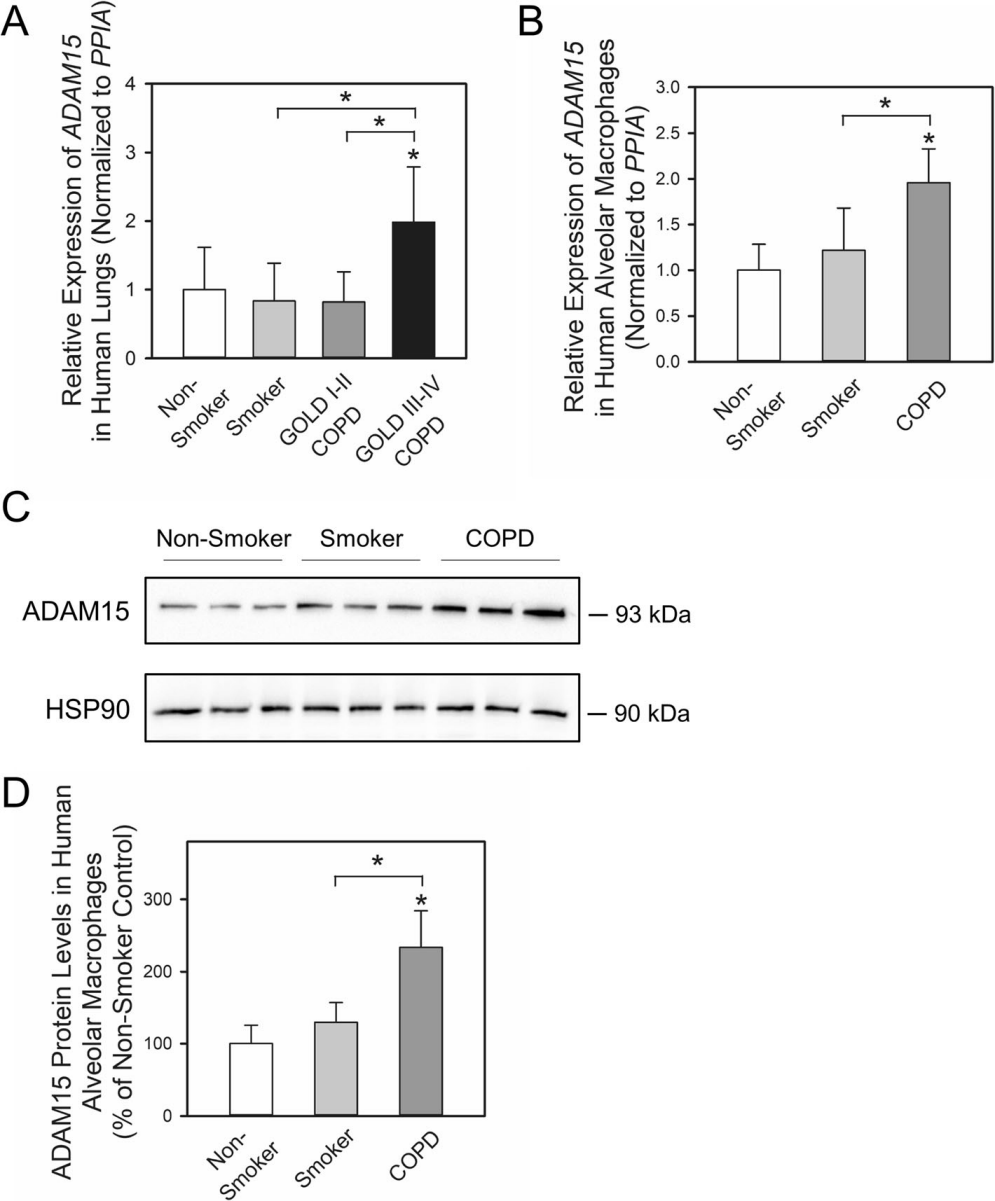
A Disintegrin and A Metalloproteinase Domain 15 (ADAM15) expression is increased in lung samples and alveolar macrophages (AMs) from patients with chronic obstructive pulmonary disease (COPD) versus controls. In **a**, *ADAM15* steady state mRNA levels were measured in lung samples from non-smokers, smokers, and patients with COPD with GOLD stage I-II and GOLD stage III-IV disease using real time RT-PCR (*n* = 17–31 subjects/group). Data are mean + SD. Data were analyzed using one-way ANOVA followed by pair-wise testing with 2 tailed Student’s t-tests. *, *P <* 0.001 versus non-smoker group or the group indicated. In **b**, *ADAM15* steady state mRNA levels were measured in AMs from non-smokers, smokers, and patients with COPD (all had GOLD stage I-III disease and 79% of these patients had GOLD stage I or II disease) in the BAL cohort using real time RT-PCR (*n* = 6–15 subjects/group). Data are mean + SD. Data were analyzed using one-way ANOVA followed by pair-wise testing with 2 tailed Student’s t-tests. *, *P <* 0.001 versus non-smoker control or the group indicated. In **c**-**d**, ADAM15 and a housekeeping control (heat shock protein 90; HSP90) were quantified in AMs from non-smokers, smokers, and patients with COPD using Western blotting and densitometry. The ADAM15 levels were normalized to HSP90 levels measured in the samples and expressed as a % of the values for the non-smoker control group. The images shown in **c** are representative of 6–9 subjects/group. **d**: Data are mean + SD (n = 6–9 subjects/group). Data were analyzed using a One-Way ANOVA followed by pair-wise testing with 2 tailed Student’s t-tests. *, *P* < 0.001 versus non-smoker control or the group indicated

Table 1 shows the demographic and clinical characteristics of the cohort from whom whole lung samples were obtained. GOLD stage III-IV patients with COPD had higher *ADAM15* mRNA levels in whole lung samples than non-smokers, smokers, and GOLD stage I-II patients with COPD (Fig. 1a). These differences remained after adjusting the *P* values for differences in age, pack-years smoking history, and current smoker status between the groups (Table 1). There were no differences in *ADAM15* mRNA lung levels among patients with COPD with GOLD stage I-II disease, smokers, and non-smokers.

### BAL samples

Table 2 shows the demographic and clinical characteristics of the BAL cohort. Ethics approval was granted to perform BAL only on patients with COPD having GOLD I-III stage disease. Most (79%) of the patients with COPD had GOLD stage I or II disease. *ADAM15* mRNA and protein levels were higher in alveolar macrophages (AMs) from patients with COPD than smokers and non-smokers (Fig. 1b-d). These differences remained after adjusting the *P* values for differences in the pack-years smoking history between the groups (Table 2). Due to the small number of subjects with GOLD III stage disease, it was not possible to relate ADAM15 transcript or protein levels in AMs to GOLD stage.

**Fig. 2:**
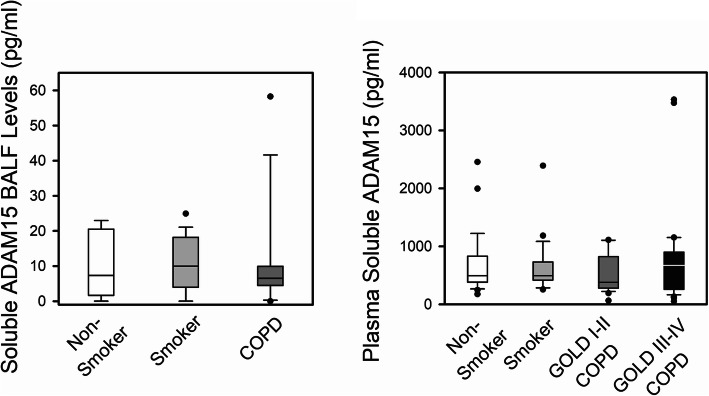
Soluble ADAM15 (sADAM15) levels in bronchoalveolar lavage fluid (BALF) and plasma samples from patients with chronic obstructive pulmonary disease (COPD), smokers, and non-smokers. **a**: Soluble ADAM15 (sADAM15) protein levels were measured in BALF samples from non-smokers (n = 6), smokers (*n* = 19), and patients with COPD (*n* = 14) using an ELISA kit. The boxes in the box-plots show the medians and 25th and 75th percentiles, and the whiskers show the 10th and 90th percentiles. Data were analyzed using a Kruskal-Wallis One-Way ANOVA followed by pair-wise testing with Mann-Whitney U tests. **b**: sADAM15 protein levels were measured in plasma samples from non-smokers (*n* = 28), smokers without COPD (*n* = 27), COPD patients with GOLD stage I-II disease (n = 27), and COPD patients with GOLD stage III-IV disease (*n* = 33) using an ELISA kit. The boxes in the box-plots show the medians and 25th and 75th percentiles, and the whiskers show the 10th and 90th percentiles. Data were analyzed using a Kruskal-Wallis One-Way ANOVA followed by pair-wise testing with Mann-Whitney U tests

Soluble forms of ADAM15 (sADAM15) have been detected in serum and synovial fluid samples from patients with rheumatoid arthritis [21], and may be generated via proteolytic shedding of ADAM15 from cell surfaces. Thus, sADAM15 levels were measured in BAL fluid (BALF) samples (Table 2) from patients with COPD and controls. BALF sADAM15 levels did not differ between patients with COPD and controls (Fig. 2a).

### Plasma samples

Table 3 shows the demographic and clinical characteristics of the plasma cohort. Soluble ADAM15 levels were measured in plasma samples from patients with COPD and controls using an ELISA kit that recognizes the ectodomain of the protein. Plasma sADAM15 levels did not differ between patients with COPD and controls (Fig. 2b).

### Immunostaining of lung sections for ADAM15

**Fig. 3:**
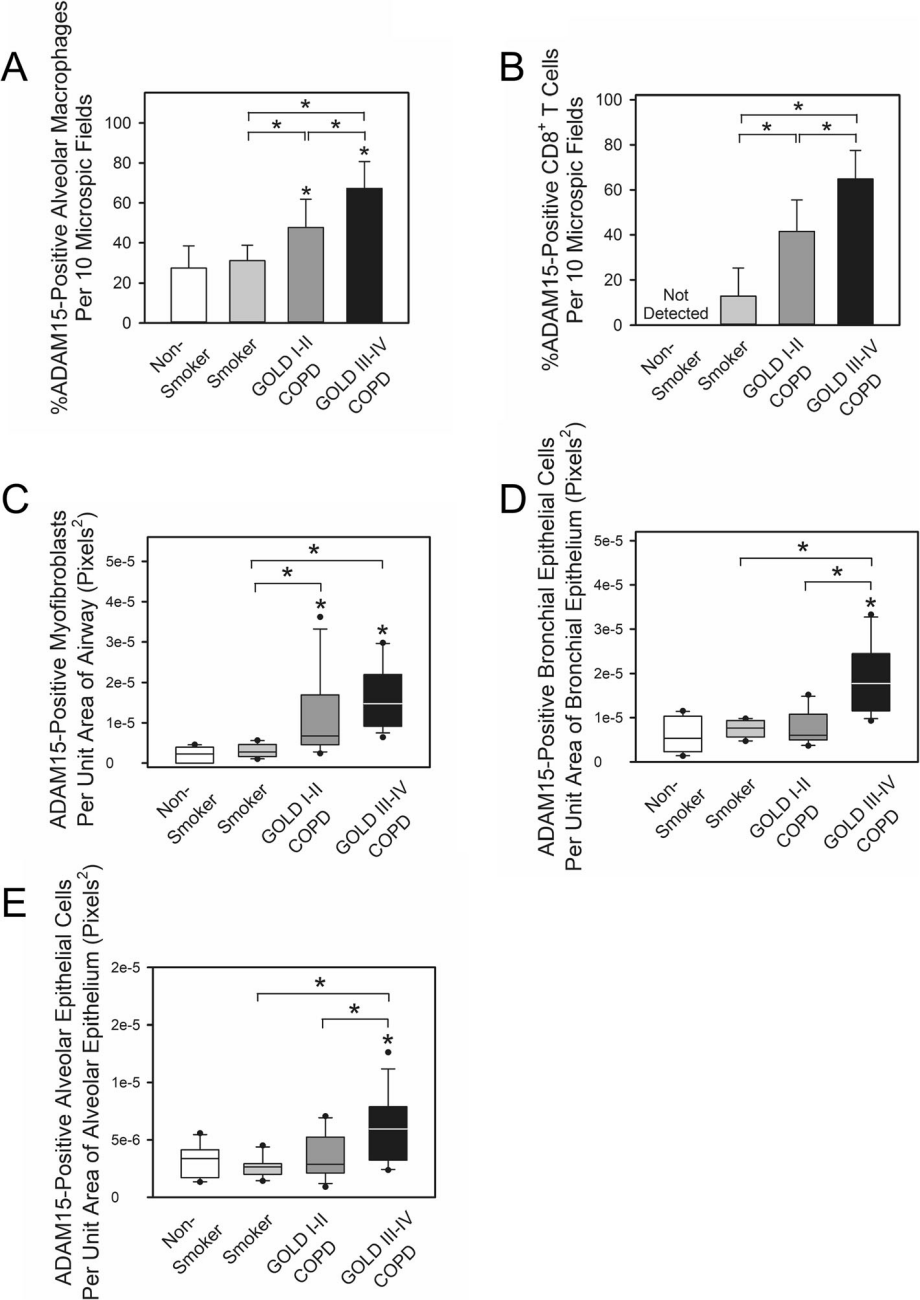
A Disintegrin and A Metalloproteinase Domain 15 (ADAM15) staining is increased in alveolar macrophages (AMs), CD8^+^ T cells, epithelial cells and α-SMC-positive cells in the lungs of patients with chronic obstructive pulmonary disease (COPD). Lung sections from 30 patients with COPD (4 had GOLD stage I, 10 had GOLD stage II, 4 had GOLD stage III, and 13 had GOLD stage IV disease), 10 smokers, and 10 non-smokers were double immunostained for ADAM15 and markers of macrophages (CD68; **a**), CD8^+^ T cells (CD8; **b**), airway cells staining positively for α-smooth muscle actin (α-SMA, a marker of myofibroblasts; **c**), bronchial epithelial cells (pancytokeratin; **d**) and alveolar epithelial cells (pancytokeratin; **e**). Lung sections that were stained with non-immune isotype-matched primary antibodies showed no staining (not shown). The percentage of ADAM15-positively stained macrophages and CD8^+^ T cells was quantified for each cell type for each subject in 10 microscopic fields. The number of ADAM15-positively stained airway α-SMA-positive cells (**c**) and bronchial epithelial cells (**d**) and alveolar epithelial cells (**e**) was quantified and normalized to the unit area (in pixels^2^) of airway wall (C), of bronchial epithelium (**d**) or of alveolar wall (**e**) using MetaMorph software. Data in **a** and **b** are mean + SD (*n* = 10–17 subjects/group). Data were analyzed using one-way ANOVA followed by pair-wise testing with 2 tailed Student’s t-tests. *, *P <* 0.003 versus non-smoker control or the group indicated. In **c-e**, the boxes in the box-plots show the medians and 25th and 75th percentiles, and the whiskers show the 10th and 90th percentiles for 10–17 subjects/group. Data were analyzed using a Kruskal-Wallis One-Way ANOVA followed by pair-wise testing with Mann-Whitney U tests. *, *P <* 0.015 versus non-smoker control or the group indicated

Lung sections from patients with COPD and controls were double immunostained for ADAM15 (using an antibody that detects the ectodomain of the protein) and markers of different cell types in the lungs to identify the cells in the lungs of patients with COPD that express ADAM15. Table 4 shows the demographic and clinical characteristics of the lung immunostaining cohort. Patients with COPD had greater ADAM15 staining in AMs (Fig. 3a and Fig. E1), CD8^+^ T cells (Fig. 3b), and α-smooth muscle actin (α-SMA)-positive cells (likely myofibroblasts) around the small airways (Fig. 3c) than non-smokers and smokers. Patients with COPD with GOLD stage III-IV disease had greater ADAM15 staining in bronchial and alveolar epithelial cells (Figs. 3d-2e) than non-smokers, smokers, and GOLD stage I-II COPD patients. ADAM15 staining was not detected in CD4^+^ T cells or CD45^+^ B cells in sections of lung from patients with COPD, smokers, or non-smokers (data not shown).

**Fig. 4:**
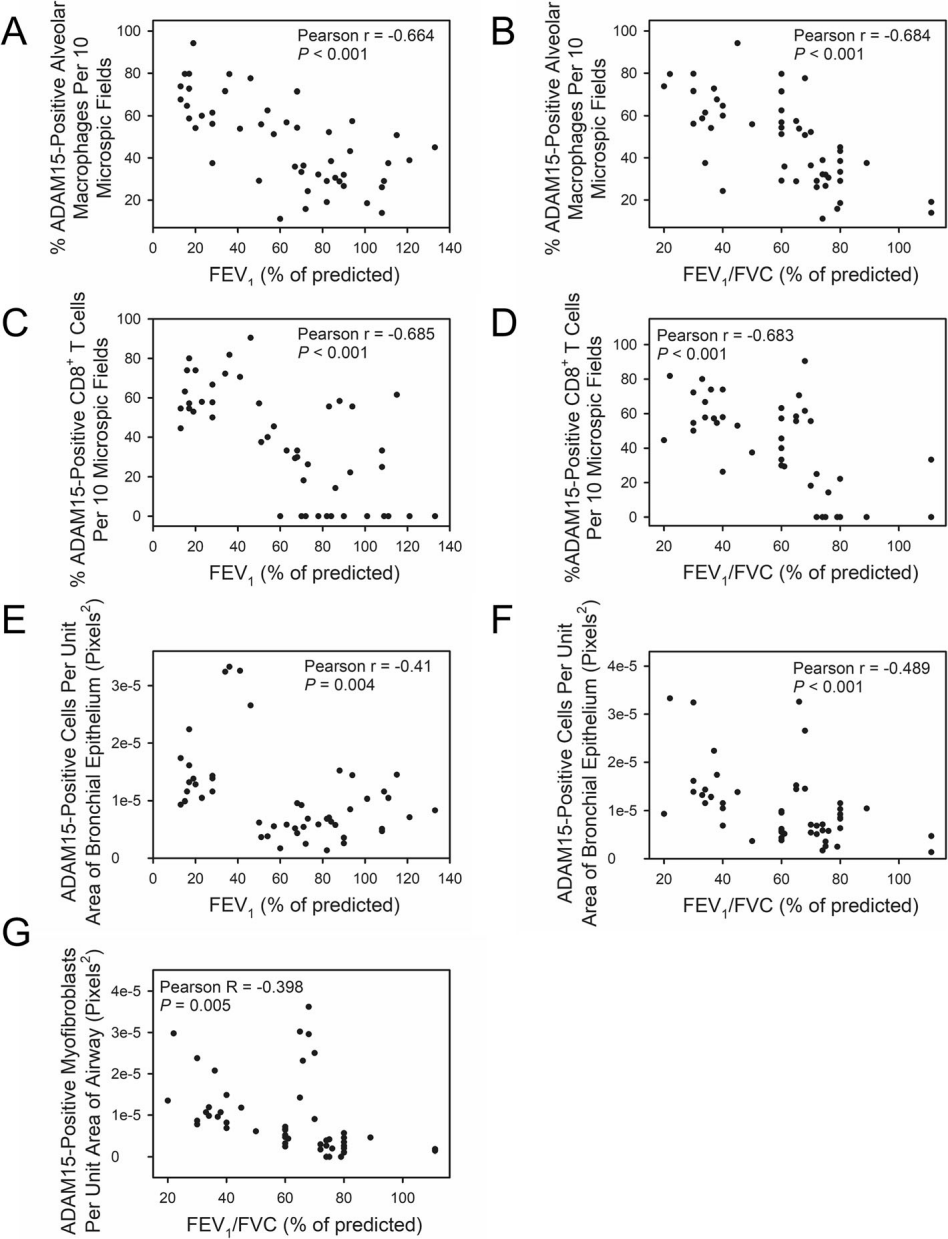
Correlations between A Disintegrin and A Metalloproteinase Domain 15 (ADAM15-positively stained) alveolar macrophages (AMs), CD8^+^ T cells, bronchial epithelial cells, alveolar epithelial cells, or α-SMC-positive airway cells in the lungs of patients with chronic obstructive pulmonary disease (COPD) and lung function parameters. Lung sections from 30 patients with COPD (4 were GOLD stage I, 10 were GOLD stage II, 4 were GOLD stage III, and 13 were GOLD stage IV), 9 smokers, and 10 non-smokers were double immunostained for ADAM15 and markers of macrophages (CD68), CD8^+^ T cells (CD8), α-smooth muscle actin; α-SMC (a marker of myofibroblasts), bronchial epithelial cells (pancytokeratin) and alveolar epithelial cells (pancytokeratin). The percentage of ADAM15-positively stained macrophages, CD8^+^ T cells, and the number of ADAM15-positively stained α-SMC-positive airway cells, bronchial epithelial cells or alveolar epithelial cells per unit area of airway wall, bronchial epithelial area, or alveolar wall area, respectively for each subject (on the y-axis) was quantified (as described in the legend to Fig. 3) and plotted against the subject’s FEV_1_ or FEV_1_/FVC values (on the x-axis). Correlations between the percentage of ADAM15-positively stained AMs and FEV_1_ percent predicted or FEV_1_/FVC percent predicted are shown in **a** and **b**, respectively. Correlations between the percentage of ADAM15-positively stained CD8^+^ T cells and FEV_1_ percent predicted and FEV_1_/FVC percent predicted are shown in **c** and **d**, respectively. Correlations between the number of ADAM15-positively stained bronchial epithelial cells per unit area of bronchial epithelium and FEV_1_ percent predicted and FEV_1_/FVC percent predicted are shown in **e** and **f**, respectively. **g** shows the correlation between the number of ADAM15-positively stained α-SMC-positive airway cells per unit area of airway wall and FEV_1_/FVC percent predicted. All data were analyzed using the Spearman Correlation test; *n* = 49 subjects in each sub-figure. *P* < 0.05 was considered to be statistically significant

**Fig. 5:**
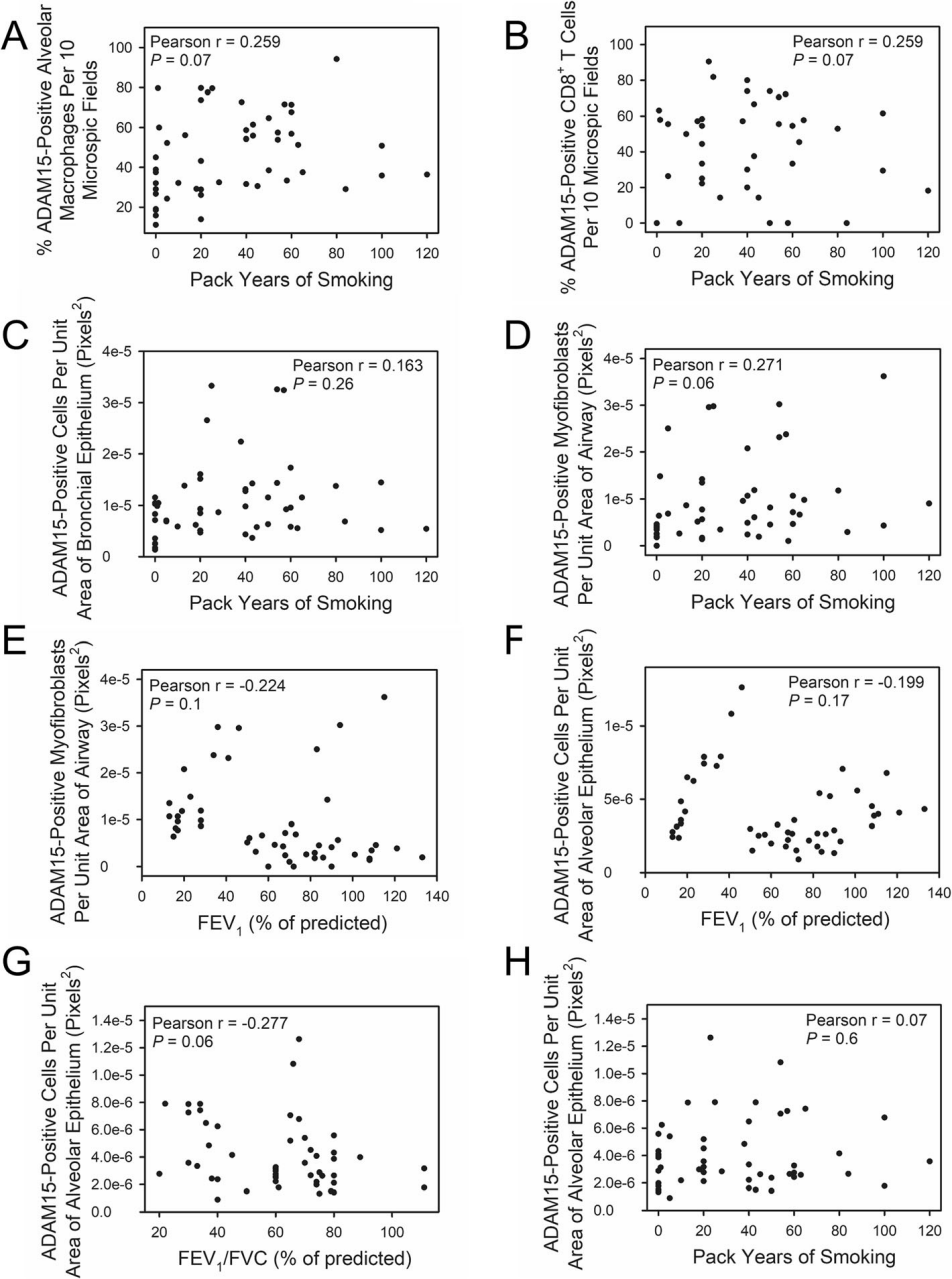
Correlations between A Disintegrin and A Metalloproteinase Domain 15 (ADAM15-positively stained) alveolar macrophages (AMs), CD8^+^ T cells, bronchial epithelial cells, alveolar epithelial cells, or α-smooth muscle actin (α-SMC)-positive airway cells and lung function or other clinical parameters. Lung sections from 31 patients with COPD (4 had GOLD stage I, 10 had GOLD stage II, 4 had GOLD stage III, and 13 had GOLD stage IV disease), 10 smokers, and 10 non-smokers were double immunostained for ADAM15 and markers of macrophages (CD68), CD8^+^ T cells (CD8), bronchial and alveolar epithelial cells (pancytokeratin), and α-SMC-positive small airway cells (likely myofibroblasts). The percentage of ADAM15-positively stained macrophages or CD8^+^ T cells in 10 microscopic fields, or the number of ADAM15-positively stained α-SMC-positive airway cells per unit area of airway wall, the number of ADAM15-positively stained bronchial epithelial cells per unit area of bronchial epithelium, or the number of ADAM15-positively stained alveolar epithelial cells per unit area of alveolar wall for each subject (on the y-axis) was quantified (as described in the legend to Fig. 3) and plotted against the subject’s pack-years of smoking history, FEV_1_, or FEV_1_/FVC (on the x-axis). **a-d**: show the correlations between the percentage of ADAM15-positively stained AMs (**a**; *n* = 50 subjects), the percentage of ADAM15-positively stained CD8^+^ T cells (**b**; n = 50 subjects), the number of ADAM15-positively stained bronchial epithelial cells per unit area of bronchial epithelium (**c**; n = 50 subjects), the number of ADAM15-positively stained α-SMC-positive airway cells per unit area of airway wall (**d**; n = 50 subjects) and pack-years of smoking history. **e** shows the correlation between the number of ADAM15-positively stained α-SMC-positive airway cells per unit area of airway wall and FEV_1_ percent predicted (n = 49 subjects including 10 non-smokers, 8 smokers and 31 patients with COPD). **f-h**: show the correlation between the number of ADAM15-positively stained alveolar epithelial cells per unit area of alveolar wall and FEV_1_ percent predicted (**f**; n = 49 subjects including 10 non-smokers, 9 smokers and 30 patients with COPD), FEV_1_/FVC percent predicted (**g**; n = 49 subjects including 10 non-smokers, 9 smokers and 30 patients with COPD) and pack-years of smoking history (**h**; n = 50 subjects). All data were analyzed using the Spearman Correlation test. *P* < 0.05 was considered to be statistically significant

ADAM15 staining in AMs (Fig. 4a-b), CD8^+^ T cells (Fig. 4c-d), and bronchial epithelial cells (Fig. 4e-f) was inversely related to FEV_1_ and FEV_1_/FVC. ADAM15 staining in AMs, CD8^+^ T cells, and bronchial epithelial cells did not correlate with pack-years smoking history (Fig. 5a-c). ADAM15 staining levels in airway α-SMA-positive cells was inversely to FEV_1_/FVC (Fig. 4g), but was not related to FEV_1_ or pack-years smoking history (Fig. 5d-e). ADAM15 staining levels in alveolar epithelial cells did not correlate with FEV_1_, FEV_1_/FVC, or pack-years smoking history (Fig. 5f-h).

### Overexpression of ADAM15 in human THP-1 cells reduces cigarette smoke extract (CSE)-induced release of cytokines and proteinases that are linked to the pathogenesis of COPD

**Fig. 6:**
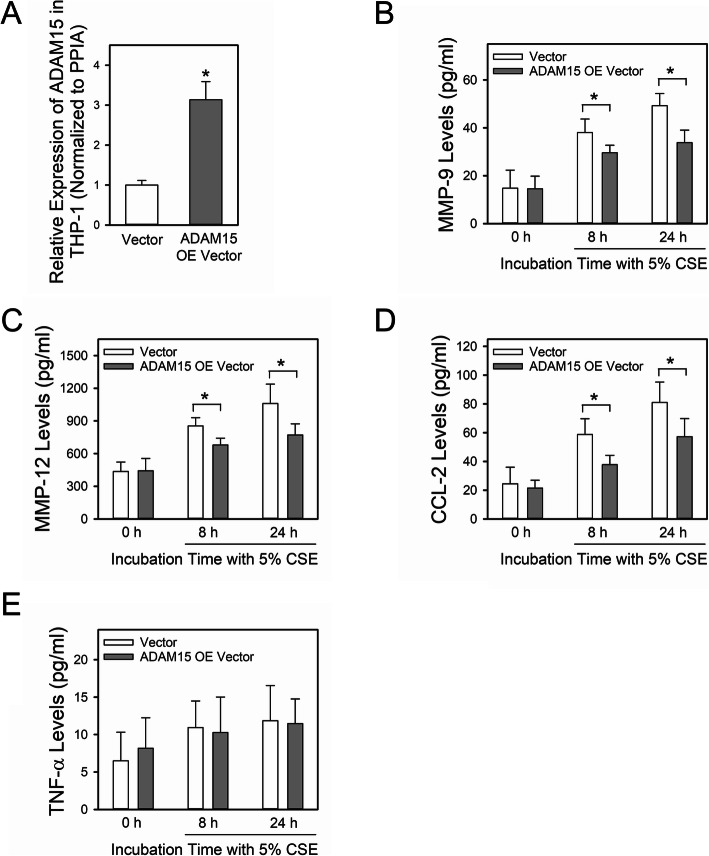
Overexpression of A Disintegrin and A Metalloproteinase Domain 15 (ADAM15) in human THP-1 cells reduces cigarette smoke extract (CSE)-induced release of cytokines and proteinases. In **a**, *ADAM15* steady state mRNA levels were measured in total cellular RNA samples isolated from differentiated THP-1 cells 24 h after they were electroporated with the pcDNA3.1-ADAM15 plasmid (ADAM15 over-expression [OE] vector) or a control vector (Vector), as described in Methods. In **b-e**, THP-1 cells were electroporated with either the ADAM15 OE or the control vector and then incubated with 5% CSE for the time indicated. Levels of MMP-9 (**b**), MMP-12 (**C**), CCL-2 (**d**) and TNF-α (**e**) protein secreted into the media were quantified using commercial ELISA kits. Data are mean + SD. Data were analyzed using a One-Way ANOVA followed by pair-wise testing with two-tailed Student’s t-tests. *, *P* < 0.05 versus the group indicated

ADAM15 was over-expressed in differentiated human THP-1 cells, as described in Methods. Robust *ADAM15* expression was detected in THP-1 cells using qRT-PCR in THP-1 cells 24 h after the cells were transduced with the vector containing the ADAM15 cDNA but not in cells treated with the control vector (Fig. 6a). When the THP-1 cells were treated with 5% CSE for up to 24 h, ADAM15 over-expression in THP-1 cells decreased their release of MMP-9 (Fig. 6b), MMP-12 (Fig. 6c) and CCL-2 (Fig. 6d) but did not affect their release of TNF-α (Fig. 6e). These data indicate that the increased ADAM15 levels detected in AMs in the lungs of patients with COPD regulates a function of AMs (release of MMPs and a cytokine) that is relevant to the pathogenesis of COPD.

## Discussion

ADAM15 has not been studied previously in COPD and little is known about its expression or activities in health or other lung diseases. To our knowledge, our study is the first to report that ADAM15 expression is increased in the lungs of patients with COPD. ADAM15 steady state mRNA and/or protein levels were increased in whole lung samples from patients with COPD and alveolar macrophages isolated from patients with COPD via BAL. Immunostaining experiments conducted on lung tissue demonstrated increased ADAM15 staining in the lungs of patients with COPD versus controls, and localized this staining to macrophages, CD8^+^ T cells, bronchial and alveolar epithelial cells, and airway α-SMC-positive cells (likely myofibroblasts). Increased ADAM15 staining in alveolar macrophages, CD8^+^ T cells, and airway α-SMC-positive cells was detected even in early stage COPD (GOLD stage I and II) whereas increased staining for ADAM15 in bronchial and alveolar epithelial cells was detected only in GOLD stage III and IV patients. ADAM15 staining in macrophages, CD8^+^ T cells, and bronchial epithelial cells was inversely related to FEV_1_ and the FEV_1_ /FVC ratio. In addition, ADAM15 staining in airway α-SMC-positive cells was inversely related to the FEV_1_ /FVC ratio but was not related to FEV_1_. These data suggest that increased ADAM15 expression in macrophages, CD8^+^ T cells, bronchial epithelial cells, and airway myofibroblasts contributes to the genesis and/or severity of airflow limitation in patients with COPD. In addition, ADAM15 over-expression in a human monocyte/macrophage-like cell line (THP-1 cells) decreased CSE-induced release of MMP-9, MMP-12, and CCL-2 by the cells, suggesting that the increased ADAM15 expression in AMs in the lungs of patients with COPD regulates the release of mediators from these cells to contribute to the disease process.

### Mechanism underlying the increased ADAM15 expression in the lungs of patients with COPD

The expression of genes in the lungs of patients with COPD generally correlates most strongly with current smoker status [22]. However, there was no clear relationship between current smoker status and *ADAM15* gene expression in the frozen lung samples as *ADAM15* expression was higher in GOLD stage III-IV patients (all of whom were former smokers) than GOLD stage I-II patients (most of whom were current smokers), and the GOLD stage I-II group had similar *ADAM15* expression as the smoker group (most of whom were former smokers). ADAM15 transcript and protein levels remained significantly higher in AMs after adjusting *P* values for differences in current smoking between patients with COPD and controls.

ADAM15 expression is upregulated in macrophages in diseased tissues other than the lung [12, 13, 23–25]. Treating macrophages with vascular endothelial growth factor [12] or fibroblast-like cells with bacterial lipopolysaccharide in vitro [26] increases ADAM15 expression by these cells. To our knowledge the effects of other mediators on macrophage *ADAM15* expression have not been examined. The lungs of patients with COPD have increased levels of pro-inflammatory mediators [4], and it is possible that these mediators drive the increase the expression of ADAM15 in leukocytes, epithelial cells, and α-SMC-positive cells in the lungs or airways of patients with COPD. *ADAM15* expression in COPD lungs could also be regulated by (epi) genetic processes. The *ADAM15* promoter contains three specificity protein-1 (Sp1)-binding sites [27], and CS increases the transcription of other genes by increasing the binding of Sp1 to the promoter regions of these other genes [28]. Whether Sp1 binding to the *ADAM15* promoter contributes to the increased *ADAM15* expression observed in COPD lungs is unclear. Single-nucleotide polymorphisms in the *ADAM15* locus are linked to rheumatoid arthritis [25], but have not linked to COPD. MicroRNAs (miRNAs) regulate gene transcription in COPD [29]. MiR-147b binds the 3′ untranslated region of the *ADAM15* mRNA in endothelial cells to reduce *ADAM15* expression [30]. Whether other miRNAs increase *ADAM15* expression in COPD lungs will be investigated in future studies.

### ADAM15 expression in leukocytes

*ADAM15* steady state mRNA levels were elevated in whole lung samples only in patients with severe or very severe COPD. We did not have a sufficient amount of whole lung sample from the subjects in the frozen lung tissue cohort in which to also measure ADAM15 protein levels. However, the data from the immunostaining cohort provided information on ADAM15 protein levels in the lung and also on ADAM15 staining in different cell types in the lung that have been implicated in the pathogenesis of COPD.

ADAM15 steady state mRNA and protein levels were increased in AMs from patients with COPD in the BAL cohort most of whom had only mild or moderately-severe disease. Immunostaining experiments performed on lung sections confirmed that ADAM15 staining was increased in lung macrophages in COPD patients versus controls and greater in patients with GOLD stage III-IV versus GOLD stage I-II disease. In addition, ADAM15 staining was increased in CD8^+^ T cells in lung sections from patients with COPD even with early stage disease. ADAM15 staining levels in both AMs and CD8^+^ T cells was inversely related to FEV_1_ and FEV_1_ /FVC. Together, these results suggest that increased ADAM15 expression in these leukocytes contributes to disease initiation and severity.

ADAM15 is known to be expressed by activated macrophages in other diseases [9], but ADAM15 was previously reported not to be expressed by CD8^+^ T cells either in healthy subjects or patients with rheumatoid arthritis [31]. To our knowledge, our study is the first to report that ADAM15 is expressed by CD8^+^ T cells. Macrophages and CD8^+^ T cells are increased in numbers in the lungs of patients with COPD and play critical roles in emphysema development in CS-exposed mice [32–34]. Macrophages promote emphysema development releasing proteinases, oxidants, and pro-inflammatory mediators. CD8^+^ T cells contribute to emphysema development by releasing perforin and granzymes that injury alveolar septal cells [34, 35], and ligands for C-X-C chemokine receptor-3 (CXCR3) which bind to this receptor on macrophages to promote macrophage activation [35].

To gain insight into the functional consequences of increased ADAM15 expression in macrophages in the lungs of patients with COPD, ADAM15 was over-expressed in differentiated THP-1 cells (a monocyte/macrophage-like cell line), the cells were exposed to CSE and the release of mediators relevant to the pathogenesis of COPD was quantified. Over-expression of ADAM15 in these cells reduced their CSE-induced release of MMP-9, MMP-12, and CCL-2. It is noteworthy that immune cell dysfunction (including AM dysfunction) is a well-recognized feature of COPD and likely contributes to colonization of the airways of COPD patients with pathogens and also to their recurrent pulmonary infection/exacerbations which contribute to lung function decline in this disease [36–38]. MMP-12 has important host defense activities (by promoting the killing of gram positive and gram negative bacteria by binding to and disrupting bacterial cell walls within the phagolysosomes of macrophages  [39]). An effective inflammatory/immune response is also required to eliminate pathogens and is induced by the robust release of chemokines such as CCL-2 from macrophages in the lungs. Thus, our ADAM15 over-expression results in THP-1 cells suggest that the increased ADAM15 levels detected in AMs in patients with COPD versus controls (and in GOLD II-IV versus GOLD I-II) is not an epiphenomenon. Rather, increased ADAM15 levels in AMs may contribute to AM dysfunction and impaired host defense against pathogens, and possibly to recurrent acute exacerbations and disease progression. The mechanism by which increased cellular ADAM15 expression may lead to AM dysfunction is not known. However, ADAM15 has an active metalloproteinase domain which could cleave cell surface receptors that are required for optimal AM activation. In addition, ADAM15 has a cytoplasmic tail that can bind intracellular proteins including Src kinases [11] which have diverse activities in regulating cell activation and survival [40].

### ADAM15 expression in lung epithelial cells

The expression or activity of ADAM15 in healthy lung epithelial cells has not been examined previously. However, transformed intestinal-derived epithelial cell lines express ADAM15 which promotes their capacity to bind to lymphocyte cell lines and increases their release of tumor necrosis factor-α [41]. In the current study, we demonstrated increased staining for ADAM15 in both bronchial and alveolar epithelial cells in the lungs of patients with COPD versus controls. Surprisingly, ADAM15 staining in bronchial (but not alveolar) epithelial cells was inversely related to FEV_1_ and FEV_1_/FVC. It is possible that bronchial epithelial-derived ADAM15 contributes to airflow obstruction by promoting the progression of the small airway disease phenotype in patients with COPD via effects on epithelial cells (such as promoting epithelial cell to mesenchymal cell transition) or increasing bronchial epithelial cell secretion or activation of mediators that promote activation of (myo) fibroblasts in the airways.

### ADAM15 expression in α-SMC-positive airway cells

*ADAM15* expression was increased in airway α-SMC-positive cells (likely myofibroblasts) in patients with COPD and was inversely related to FEV_1_/FVC. Fibroblasts in the airways of patients with COPD are resistant to CS-induced apoptosis [42]. ADAM15 has been reported to increase the migration and survival of human fibroblasts. Fibroblast-derived ADAM15 promotes an anti-apoptotic phenotype in fibroblasts in the joints of patients with rheumatoid arthritis by activating the Src/focal adhesion kinase pathway [14]. In addition, silencing the expression of ADAM15 in fibroblast-like synoviocytes reduces their migration and release of mediators and MMPs [26], and a number of MMPs have pro-fibrotic activities [43]. Thus, increased ADAM15 expression in airway myofibroblasts may promote small airway fibrosis in patients with COPD by increasing the activation and survival of (myo) fibroblasts and/or their release of pro-fibrotic mediators.

Soluble ADAM15 was detected in BALF and plasma samples and are likely generated by shedding of surface-bound ADAM15 from leukocytes and epithelial cells by the active site of ADAM15 or other proteinases. However, sADAM15 levels in plasma and BALF did not differ between the patients with COPD and controls indicating that sADAM15 levels in these compartments are not likely to be useful as diagnostic or prognostic biomarkers for COPD.

This study has several limitations. The study was a cross-sectional analysis of samples from human cohorts that were small in size. In addition, the lung tissue, and BAL and plasma samples were obtained from different subjects and the BAL cohort was relatively small in size. We were not able to relate ADAM15 expression in AMs to COPD severity as the ethics committee did not approve the recruitment of patients with GOLD stage IV disease for the BAL procedure, and recruitment of patients with GOLD stage III disease was limited. However, data from the immunostaining cohort confirmed the data from the BAL cohort that ADAM15 levels are increased in AMs from patients with COPD versus controls and also demonstrated that the percentage of AMs staining positively for ADAM15 was inversely related to both percent predicted FEV_1_ and FEV_1_/FVC. Nevertheless, our results should be confirmed in larger human cohorts, and ADAM15 expression in different samples from the same subjects should be measured. Future studies will determine whether ADAM15 regulates the functions of other leukocytes, epithelial cells, and myofibroblasts in the lungs that contribute to the pathogenesis of COPD. In addition, longitudinal studies of ADAM15 expression in different cells in the lungs of patients with COPD are needed to determine whether ADAM15 expression levels in individual cells types is related to COPD progression.

## Conclusions

We have strongly linked increased expression of ADAM15 in the lung to human COPD for the first time. In addition, as increased ADAM15 expression in lung macrophages, CD8^+^ T cells, and bronchial epithelial cells was related to the severity of airflow obstruction, these results suggest that the increased ADAM15 expression in these cells contributes to the genesis and or progression of COPD. Whether ADAM15 expression levels can serve as a prognostic biomarker for COPD or whether strategies that regulate lung levels of ADAM15 have therapeutic potential in COPD remain to be determined. Future studies will evaluate the contributions of ADAM15 to the pathogenesis of COPD using gene-targeted mice in murine models of COPD.

## Supplementary information

**Additional file 1.**

## Data Availability

All reagents used in this manuscript are commercially available. The datasets used and/or analyzed during the current study are available from the corresponding author on reasonable request.

## Abbreviations

ADAM15: A disintegrin and metalloproteinase domain-15
AMs: Alveolar macrophages
BAL: Bronchoalveolar lavage
BALF BAL: Fluid
CD: Cluster of differentiation
COPD: Chronic obstructive pulmonary disease
CS: Cigarette smoke (CS)
CXCR3: C-X-C Chemokine receptor-3
ELISA: Enzyme-linked immunosorbent assay
FEV_1_: Forced expiratory volume in 1 s
FVC: Forced vital capacity
GOLD: Global Initiative for obstructive lung disease
MMP: Matrix metalloproteinases
mRNA: messenger ribonucleic acid
PanCK: Pancytokeratin
PPIA: Peptidylprolyl isomerase A
RIPA: Radioimmunoprecipitation assay
RT-PCR: Reverse transcription polymerase chain reaction
α-SMC: α Smooth muscle actin
Sp1: Specificity protein-1
