# Supplementary material for: ADAM15 expression is increased in lung CD8+ T cells, macrophages, and bronchial epithelial cells in patients with COPD and is inversely related to airflow obstruction
Source: Respir Res. 2020 Jul 16;21:188. doi: 10.1186/s12931-020-01446-5 (PMC7364636; doi:10.1186/s12931-020-01446-5)
Supplement: Supplementary file 1 — Additional file 1. [file 12931_2020_1446_MOESM1_ESM.docx]

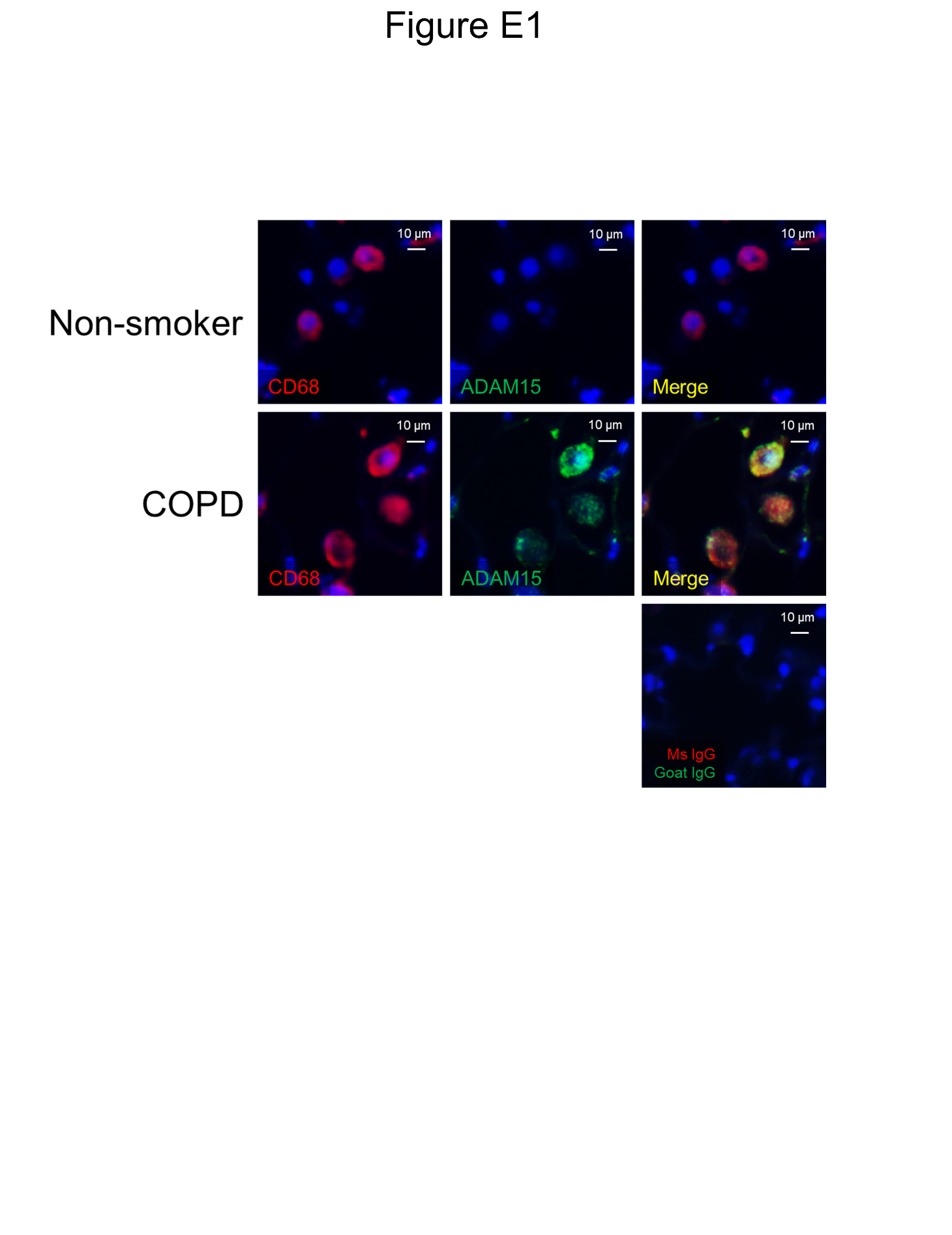
***Figure E1: A Disintegrin and A Metalloproteinase Domain 15 (ADAM15) staining is increased in alveolar macrophages (AMs) in lungs from patients with chronic obstructive pulmonary disease (COPD)***. Lung sections from 30 patients with (4 had GOLD stage I, 10 had GOLD stage II, 4 had GOLD stage III, and 13 had GOLD stage IV), 10 smokers, and 10 nonsmokers were immunostained with a red fluorophore for a marker of macrophages (CD68), and a green fluorophore for ADAM15. The nuclei were counterstained blue using 4′,6-diamidino-2-phenylindole and the sections were examined using epifluorescence microscopy. The first two rows of the figure show a representative image of a lung section from one patient with COPD and one control (a non-smoker) is shown that were double stained for CD68 (left panels) and ADAM15 (middle panels) is shown along with the merged images (right panels) are shown. The bottom row shows a representative image of a lung section from a patient with COPD that was stained with non-immune isotype matched control antibodies (murine [Ms] IgG or goat IgG.
